# Use of Hypoxic Respiratory Challenge for Differentiating Alzheimer’s Disease and Wild-Type Mice Non-Invasively: A Diffuse Optical Spectroscopy Study

**DOI:** 10.3390/bios12111019

**Published:** 2022-11-15

**Authors:** Myeongsu Seong, Yoonho Oh, Hyung Joon Park, Won-Seok Choi, Jae Gwan Kim

**Affiliations:** 1 School of Information Science and Technology, Nantong University, Nantong 226019, China; 2 Research Center for Intelligent Information Technology, Nantong University, Nantong 226019, China; 3 Department of Biomedical Science and Engineering, Gwangju Institute of Science and Technology, Gwangju 61005, Republic of Korea; 4 School of Biological Sciences and Technology, College of Natural Sciences, College of Medicine, Chonnam National University, Gwangju 61186, Republic of Korea; 5 Department of Biochemistry, University of Washington, Seattle, WA 98195, USA

**Keywords:** Alzheimer’s disease, diffuse optical spectroscopy, hemodynamic measurements, hypoxic respiratory challenge, machine learning

## Abstract

Alzheimer’s disease is one of the most critical brain diseases. The prevalence of the disease keeps rising due to increasing life spans. This study aims to examine the use of hemodynamic signals during hypoxic respiratory challenge for the differentiation of Alzheimer’s disease (AD) and wild-type (WT) mice. Diffuse optical spectroscopy, an optical system that can non-invasively monitor transient changes in deoxygenated (ΔRHb) and oxygenated (ΔOHb) hemoglobin concentrations, was used to monitor hemodynamic reactivity during hypoxic respiratory challenges in an animal model. From the acquired signals, 13 hemodynamic features were extracted from each of ΔRHb and −ΔOHb (26 features total) for more in-depth analyses of the differences between AD and WT. The hemodynamic features were statistically analyzed and tested to explore the possibility of using machine learning (ML) to differentiate AD and WT. Among the twenty-six features, two features of ΔRHb and one feature of −ΔOHb showed statistically significant differences between AD and WT. Among ML techniques, a naive Bayes algorithm achieved the best accuracy of 84.3% when whole hemodynamic features were used for differentiation. While further works are required to improve the approach, the suggested approach has the potential to be an alternative method for the differentiation of AD and WT.

## 1. Introduction

Dementia is one of the most devastating brain diseases and has various causes. The most common cause of dementia is Alzheimer’s disease (AD) [1]. The prevalence of AD is expected to keep increasing as life expectancy increases. Generally, AD is characterized by the accumulation of β-amyloid and tau protein in the brain [1,2,3,4,5]. While AD results in severe damage to the brain, which deteriorates the life of the patient and the patient’s family, there is no viable cure for AD at present. However, early diagnosis of AD is still beneficial for future planning, participation in clinical trials, and delaying the progress of the disease by regulating the patient’s lifestyle [1]. For the diagnosis of AD, various methods are used. The most conventional diagnostic methods are cognitive and behavioral tests [1]. Other than cognitive and behavioral tests, blood sampling and brain imaging have been used to diagnose AD. Among imaging methods, monitoring of β-amyloid [2,3] and tau protein [4,5] using positron emission tomography (PET) has been performed. Blood oxygenation level-dependent (BOLD)-functional magnetic resonance imaging (fMRI), a type of MRI-based technique, has also been used to investigate the differences between control, mild cognitive impairment (MCI), and AD [6]. However, the use of PET and MRI is usually costly. As an alternative approach, optical techniques, solely or combined with other techniques, have been utilized to characterize AD. Among various optical techniques, diffuse optical spectroscopy (DOS), also known as near-infrared spectroscopy (NIRS) in biomedical engineering, is frequently used in AD studies due to its relatively high temporal resolution, low cost, portability, and non-invasiveness. In most NIRS-based AD studies, experimental protocols, including the verbal fluency test [7,8,9,10,11,12], N-back test (including 1-back) [10,11,12,13], oddball test [11,12], and Stroop test [10], are used as stimuli to the brain. Such approaches aim to test neurovascular coupling to hypothesize differences in the responses of an AD group and a normal group. However, the applicability of such experimental protocols may be affected by the educational level of an individual.

Monitoring the cerebrovascular reactivity (CVR) caused by supplying breathing gas or using specific breathing methods is another approach to investigating various brain diseases, including migraine [14], traumatic brain injury [15], epilepsy [16], and cerebral small vessel disease [17]. In the meantime, some animal studies have used breathing gas challenges supplying 100% O_2_ gas or carbogen gas (95% O_2_ gas balanced by 5% CO_2_ gas) to differentiate between 3xTg, a type of AD animal model, and wild-type (WT) animals [18,19]. However, these studies did not test the use of breath-holding as a means to induce the CVR. Similarly, a spatial frequency domain imaging system, which is used in these studies, requires excision of the scalp, which cannot be easily performed on human subjects.

While many AD studies using optical techniques performed relatively simple analyses including statistical tests and comparison of signal correlations [7,8,9,11,13], thanks to the rapid development of artificial intelligence (AI) techniques such as machine learning (ML) and deep learning (DL), recent AD studies using optical techniques tend to perform more advanced analyses of acquired signals [10,12]. It is well known that AI techniques extract ‘hidden’ information that cannot be found by conventional analytical methods, thus it is believed that a wide range of medical fields will benefit from adopting AI techniques.

In this work, to explore an alternative non-invasive, simple, and cost-effective approach for diagnosing AD, preliminary animal experiments were performed during the hypoxic respiratory challenge, an intervention that mimics breath-holding. During the experiments, a DOS system was used to monitor the CVR of AD and WT. In the meantime, Monte Carlo simulations were performed to show the possibility of non-invasive brain signal measurement using the DOS system. Statistical analysis and machine learning (ML) algorithms were used to confirm the feasibility of the suggested approach and the practicality of the protocol for differentiating AD and WT.

Contributions of this study include: (1) testing hypoxic respiratory challenge in AD and WT by measuring hemodynamic signals using the DOS system; (2) showing the usefulness of extracted hemodynamic features, which are based on methods suggested in previous breast cancer studies, in the differentiation of AD and WT; (3) identifying some hemodynamic features that can differentiate AD and WT statistically significantly; (4) testing 15 ML algorithms for hemodynamic features of CVR caused by hypoxic respiratory challenge for classification of AD and WT.

## 2. Materials and Methods

### 2.1. Animal Model and Preparation

**Figure 1:**
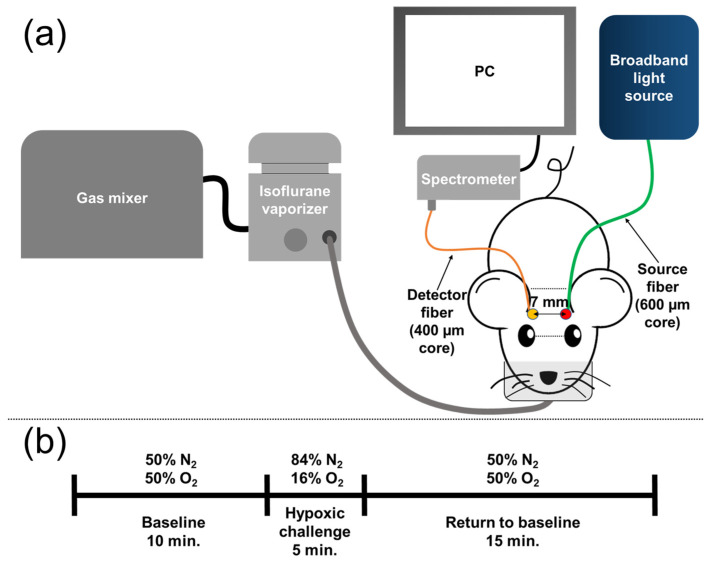
(**a**) Schematic of the animal experimental setup, (**b**) protocol of gas intervention.

We used 12-month-old male 5xFAD animals (AD, *n* = 6) and age-matched wild-type animals (WT, *n* = 6) in this study. The 5xFAD model is a genetically-modified mouse model that co-expresses 5 mutations of familial Alzheimer’s disease (FAD) [20,21,22,23], and develops accumulation of β-amyloid in a relatively short time. As a result, the model shows the characteristics (amyloid pathology and memory impairment) of FAD in a shorter time in comparison to other Alzheimer’s models. To induce anesthesia, each animal was initially placed in an induction chamber filled with 5% isoflurane mixed with 50% O_2_ gas balanced by N_2_ gas. After induction, the anesthesia was maintained with 1.5% isoflurane supplied through a nose cone (Figure 1a). The mouse head was fixed using a stereotaxic frame (Customized stereotaxic frame, Digitaxis). To prevent eye dryness, an eye ointment was applied to both eyes of the animal. The fur on the head and the thigh was shaved and depilated to minimize the influence of scattering in the DOS and pulse oximeter measurements, respectively.

### 2.2. Behavioral Tests

Before proceeding with the measurements of cerebral hemoglobin concentration, novel object recognition and Morris water maze tests were performed to confirm the AD-related behavioral changes in 5xFAD mice when the animals were 6–7 months old (5xFAD: *n* = 6; WT: *n* = 6).

#### 2.2.1. Novel Object Recognition Test

The novel object recognition test consists of training and test phases. Mice were placed for 10 min in the open-top box (40 × 40 × 40 cm^3^) where two identical objects were located during the training phase. The test phase started 24 h after the training phase. Mice were returned to the box with one novel object and the identical object used in the training phase and then explored the objects for 10 min. The preference index was calculated as the time to explore the novel object, which is expressed as ‘a’, divided by the time to explore both objects, which is expressed as ‘a + b’ (% preference index = {a/(a + b)} × 100 (%)).

#### 2.2.2. Morris Water Maze Test

The Morris water maze test was performed over 4 trials per day for 4 consecutive days to test spatial memory. A circular test pool (114 cm in diameter, 25 cm in height) was filled with opaque water using non-toxic paint and was maintained at 25 °C. For all sessions over 4 days, a clear escape platform (15 × 10.5 cm^2^) was immersed 1.5 cm below the water surface in a fixed position in the pool. In each trial, mice were randomly placed facing the wall in one of the three quadrants of the pool except for the quadrant containing the escape platform. Mice were allowed to swim to find the escape platform for 60 s. If the mice could not find the escape platform within 60 s, we guided the mice to the platform. The animals were allowed to stay on the platform for 10 s. The latency time from the starting point to the escape platform was measured for all trials.

### 2.3. Diffuse Optical Spectroscopy for Cerebral Hemoglobin Concentration Measurement

Figure 1 shows a schematic of the system. The DOS system consisted of a broadband light source (HL-2000-HP, Ocean Optics, Orlando, FL, USA) with a 600-μm multi-mode fiber (M29L02, numerical aperture (N.A.): 0.39, Thorlabs, Newton, NJ, USA) and a spectrometer (custom USB4000, wavelength: 470–1000 nm, Ocean Optics, Orlando, FL, USA) with a 400-µm multi-mode fiber (M79L01, N.A.: 0.39, Thorlabs, Newton, NJ, USA). A source-detector separation was set to 0.7 cm to balance the signal stability, which was determined by comparing the maximum intensity and the noise level, as well as the signal acquisition rate, which is inversely proportional to the integration time. The expected probing depth of the system was approximately 0.35 cm based on the diffusion theory, which was deep enough to reach the depth of the brain. Even though the overall signal-to-noise ratio may increase as the source-detector separation becomes shorter, a shorter separation is not considered due to the multi-layer nature of the brain. Specifically, the decrease of the source-detector separation will enhance the signal sensitivity on the extracerebral tissues, including the scalp and the skull, and simultaneously diminish the signal sensitivity on the brain. Thus, the source-detector separation should be long enough to achieve high enough sensitivity on the CVR. The validity of the acquisition of the signals from the brain is explored using Monte Carlo simulation (Section 2.6 and Section 3.2). DOS data were collected using a bundle software from Ocean Optics (Spectrasuite, Ocean Optics, Orlando, FL, USA). The sampling frequency of DOS varied depending on the integration time of the spectrometer since the animals showed different peak signal intensities. The integration time was set to have the maximum intensity value 3 or 4 times higher than the dark signal intensity, which resulted in 1 Hz to 3 Hz of the sampling frequency. The collected DOS raw data were saved for offline processing.

### 2.4. Signal Acquisition

The measurement was performed non-invasively, thus the bregma-lambda coordinate points could not be used. Instead, the DOS probe was placed between the horizontal line of the eyes and the horizontal line of the ears to acquire DOS signals from the same position. The probe was placed on the head of the mouse by the same person so that the position and pressure given to the head by the probe could be as constant as possible. Figure 1a shows the orientation of the source and detector fibers. Figure 1b shows the experimental protocol of the breathing gas challenge used in this work. The total measurement took 30 min. We supplied 50% O_2_ gas balanced by N_2_ gas for 10 min for the baseline measurement. After the baseline measurement, 16% O_2_ gas balanced by N_2_ gas was supplied for 5 min as the hypoxic respiratory challenge. The hypoxic respiratory challenge was used to mimic breath-holding. After the hypoxic respiratory challenge, 50% O_2_ gas balanced by N_2_ gas was supplied again for 15 min. The gas modulation was performed using a gas mixer. During the measurement, an automatic temperature controller with a rectal probe (Temperature controller, RWD Life Science, Shenzhen, Guangdong, China) maintained the core temperature of the animal as 37 °C. To ensure the safety of the animal, a pulse oximeter (Mouse Ox, Starr Inc., Oakmont, PA, USA) and a patient monitoring device (B40, GE Healthcare, Chicago, IL, USA) were used to monitor heart rate, oxygen saturation, and the respiratory function of each animal during the measurement.

Following the principles of the 3Rs (replacement, reduction, and refinement) in animal experiments (nc3rs.org.uk), to minimize the usage of animals, each animal was measured 5 to 6 times on different days. The study was approved by the Institutional Animal Care and Use Committee (IACUC) of the Gwangju Institute of Science and Technology (Protocol number: GIST-2017-065) and the Chonnam National University (protocol number: CNU IACUC-YB-2018-08).

### 2.5. Modified Beer-Lambert’s Law

To extract relative hemoglobin concentration from the DOS raw data, the modified Beer–Lambert law (MBLL) was used [24,25,26]. Equation (1) shows MBLL with wavelengths of 780, 808, and 820 nm:

(1) $$\left(\begin{array}{c} \Delta RHb\\ \Delta OHb \end{array}\right)=\frac{1}{d\cdot DPF}{\left(\begin{array}{cc} \varepsilon_{RHb}^{780\;\mathrm{nm}} & \varepsilon_{OHb}^{780\;\mathrm{nm}}\\ \varepsilon_{RHb}^{808\;\mathrm{nm}} & \varepsilon_{OHb}^{808\;\mathrm{nm}}\\ \varepsilon_{RHb}^{820\;\mathrm{nm}} & \varepsilon_{OHb}^{820\;\mathrm{nm}} \end{array}\right)}^{-1}\left(\begin{array}{c} \Delta OD^{780\;\mathrm{nm}}\\ \Delta OD^{808\;\mathrm{nm}}\\ \Delta OD^{820\;\mathrm{nm}} \end{array}\right)$$

where RHb and OHb denote deoxygenated and oxygenated hemoglobin, respectively, ΔRHb and ΔOHb denote the relative RHb and OHb changes, respectively, d denotes a source –detector separation (7 mm in this study), DPF denotes the differential path length factor, $\varepsilon_{RHb}^{\lambda }$ and $\varepsilon_{OHb}^{\lambda }$ denote the extinction coefficient of RHb and OHb at the different wavelengths, respectively, and $\Delta OD^{\lambda }$ ($=log_{10}\left(I_{t}^{\lambda }/I_{0}^{\lambda }\right)$) denotes the transient optical density at the different wavelengths. $I_{t}^{\lambda }$ and $I_{0}^{\lambda }$ are the transient and baseline intensity at the different wavelengths, respectively. The average intensity of the first 20 s signals at the different wavelengths was considered as $I_{0}^{\lambda }$. The $\varepsilon_{RHb}$ and $\varepsilon_{OHb}$ at different wavelengths are from a web page hosted by S. Prahl and S. Jacques [27]. The unit of hemoglobin concentration was set to be mM/DPF, which is equivalent to setting DPF as 1 at different wavelengths [24]. Note that considering DPF as a part of the unit of hemoglobin concentration is one of the widely accepted approaches [12,24,25,28]. In this study, 808 nm was selected because it is known as one of the isosbestic points of the extinction coefficients of RHb and OHb [29]. Meanwhile, 780 nm and 820 nm were selected to reflect the different light absorption tendencies between RHb and OHb. Still, other optical wavelengths can be used depending on experimental conditions. Owing to the existence of a non-square matrix of the extinction coefficients in MBLL, the Moore–Penrose pseudoinverse was used to get the inverse of the non-square matrix [26,30].

### 2.6. Monte Carlo Simulation of Probing Depth

The Monte Carlo simulation is widely used for the simulation of light propagation through various types of media. To validate the probing depth of the DOS system with the 7 mm source-detector separation, a set of Monte Carlo simulations was performed using a MCXLAB toolbox [31]. For simplicity, a three-layer (scalp, skull, and brain), 2D flat slab with a dimension of 10 × 20 mm^2^ (*x*-axis × *z*-axis) was assumed. Table 1 shows the dimensional and optical properties of the scalp, skull, and brain used in the simulation. Due to the unavailability of appropriate absorption coefficients of the scalp, following the work of S. Y. Lee et al. [32], estimated constituents of absorbers of the human extracerebral tissue that treat the scalp and the skull as a single tissue layer [32,33] were used to get absorption coefficients of the scalp at desired wavelengths. In one simulation, the source was set to have a size of 600 µm and a NA of 0.39 to mimic the source fiber in the measurements. In another simulation, the source was set to have a size of 400 µm and a NA of 0.39 to mimic the detector fiber in the measurements. Each simulation was performed by launching 10^7^ photons. Two separate simulations performed with the two sources were set to have a 7 mm distance to mimic the 7 mm source-detector separation. Element-wise production was performed on the fluence results of the two simulations to get depth sensitivity maps. Note that this approach is commonly used to get the depth sensitivity map [34,35].

### 2.7. Extraction of Hemodynamic Features

To quantify differences between AD and WT, quantitative hemodynamic features were adopted from breast cancer studies that used diffuse optical tomography—an optical method that can acquire 3D absorption and scattering images of biological tissues—and dynamic contrast-enhanced MRI [41,42]. Originally, the features were suggested to quantify the hemodynamic response of the breast during hypoxic respiratory challenge for diagnosing breast cancer and monitoring neoadjuvant chemotherapy efficacy. While we investigated AD in this study because the study also aimed to quantify hemodynamic response caused by hypoxic respiratory challenge, the hemodynamic features used in the breast cancer studies were adopted with a slight modification. Six hemodynamic features derived via simple arithmetic calculation are shown below [41,42]:

(2) $$S_{min}=\min\left\{S\left(t\right)\right\};\;\;\;t:600-700\;s$$

(3) $$S_{max}=\max\left\{S\left(t\right)\right\};\;\;\;\;\;t:900-1000\;s,$$

(4) $$IE=\left(S_{max}-S_{min}\right)/S_{max},$$

(5) $$PIE=\left(S_{PostBH30sec}-S_{max}\right)/S_{max},$$

(6) $$m_{rise}=\left(S_{max}-S_{min}\right)/\left(T_{max}-T_{min}\right),$$

(7) $$m_{fall}=\left(S_{max}-S_{PostBH30sec}\right)/\left(T_{max}-T_{PostBH30sec}\right),$$

where $S\left(t\right)$ denotes the signal as a function of time, $S_{min}$ denotes the minimum of the signal within 600–700 s, $S_{max}$ denotes the maximum of the signal within 900–1000 s (after the end of hypoxic respiratory challenge), IE denotes initial enhancement, PIE denotes post-initial enhancement, $S_{PostBH30sec}$ denotes the amplitude of the signal at 930 s (30 s after the end of hypoxic respiratory challenge), $m_{rise}$ denotes the slope of the rise within $T_{max}$ and $T_{min}$, $T_{max}$ and $T_{min}$ denote the corresponding times of $S_{max}$ and $S_{min}$, respectively, $m_{fall}$ denotes the slope of the drop within $T_{max}$ and $T_{PostBH30sec}$, and $T_{PostBH30sec}$ denotes the corresponding time of $S_{PostBH30sec}$.

**Figure 2:**
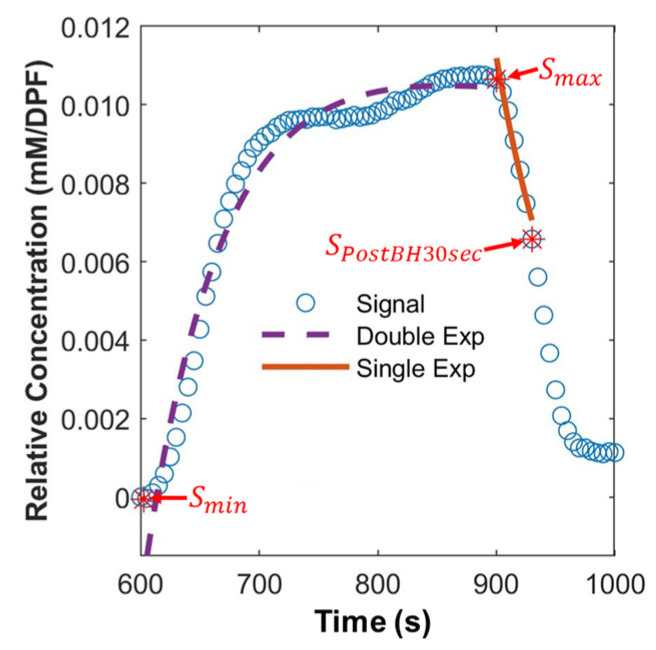
Example of hemodynamic features extraction (from the grand average of ΔRHb of the AD group). Blue dots indicate the signal. $S_{min}$: the smallest amplitude of the signal between 600 and 700 s; $S_{max}$: the largest amplitude of the signal between 900 and 1000 s; $S_{PostBH30sec}$: the amplitude at 930 s (30 s after the end of hypoxic respiratory challenge). A purple dashed line indicates the result of the double exponential fitting to the signal between 600 and 900 s. An orange line indicates the result of the single Exponential fitting to the signal between 900 and 930 s.

The other four parameters are derived via nonlinear fitting of the signal to the equations shown below [41,42,43]:

(8) $$S\left(t\right)=A_{rise,1}\times e^{\left(q_{rise,1}\right)t}+A_{rise,2}\times e^{\left(q_{rise,2}\right)t};\;\;\;\;t:600-900\;s,$$

(9) $$S\left(t\right)=A_{fall}\times e^{\left(q_{fall}\right)t};\;\;\;t:900-1000\;s,$$

where $A_{rise,\;1}$ and $A_{rise,2}$ denote the amplitudes of the rise of the first and second exponentials, respectively, e denotes the exponential function, $q_{rise,1}$ and $q_{rise,2}$ denote the rate of the rise of the first and second exponentials, respectively, $A_{fall}$ denotes the amplitude of the washout rate, and $q_{fall}$ denotes the rate of the drop. Figure 2 shows the example of the extraction of the hemodynamic features.

All of the signals were detrended, up-sampled to 10 Hz, and then smoothed using a moving average filter (window size: 500) before extracting the features described above. In the case of ΔRHb, the features were calculated directly. In the case of ΔOHb, the features were calculated using the negative of the signal (−ΔOHb) due to its trend toward the negative during hypoxic respiratory challenge. Note that the references of the hemodynamic features used signals at 15 s after the end of hypoxic respiratory challenge for calculating some features (e.g., $S_{PostBH15sec}$ and $T_{PostBH15sec}$); however, here signals at 30 s after the end of hypoxic respiratory challenge were used due to the use of anesthesia that may alter the vascular response. For Equation (8), the double exponential was used in this study due to the tendency of the resultant signals while the references used the single exponential.

The features in Equations (8) and (9) ($A_{rise1}$, $A_{rise2}$, $q_{rise1}$, $q_{rise2}$, $A_{fall}$, and $q_{fall}$) were calculated using *fminsearch*, a MATLAB built-in function for nonlinear fitting (MATLAB R2019b, MathWorks, Natick, MA, USA). Among the whole data set, time-series data with issues, including system issues, unstable measurements, and different experimental conditions, were totally excluded and not used throughout the study.

### 2.8. Statistical Analysis

Before performing the statistical analysis, normality tests were performed for each hemodynamic parameter using the Jarque–Bera test [44]. When a hemodynamic parameter of both AD and WT passed the normality test, the two-sample *t*-test [45] was used to perform the statistical analysis. Otherwise, the Wilcoxon rank-sum test [46] was used to perform the statistical analysis. In this work, a *p*-value lower than 0.05 was considered to be statistically significant. Normality tests and statistical analysis were performed using built-in functions in MATLAB (MATLAB R2019b, MathWorks, Natick, MA, USA).

### 2.9. Machine Learning (ML)-Based Classification

ML is a widely utilized technique for regression and classification. To further demonstrate the feasibility of the suggested method for differentiating AD and WT, we performed ML-based classification. Among various ML toolboxes, PyCaret was used because PyCaret is a handy library that offers multiple widely used ML algorithms [47]. ML algorithms used in the study include logistic regression [48], ridge classifier [49], linear discriminant analysis [50,51], K-nearest neighbor classifier [49], support vector machine [52], naive Bayes [49], decision tree classifier [53], gradient boosting algorithm [54], light gradient boosting machine [54], random forest classifier [55], quadratic discriminant analysis [51], extreme gradient boosting [54], AdaBoost classifier [56], extra trees classifier [57], and CatBoost classifier [54]. Note that various ML algorithms have been tested since there is no fixed guidance or solution that determines which algorithm works best for a specific problem. A total of 80% of the data were used as training data to fine-tune each algorithm and 20% of data were used to evaluate the fine-tuned algorithm. K-fold with a fold number of 32 was used due to the limited datasets of the study. For each fold, data were shuffled. Data imbalance was mitigated using a synthetic minority over-sampling technique (SMOTE) [47,58]. Outliers, which were replaced as a not-a-number (NaN) during the extraction of the hemodynamic parameters in Section 2.7, were imputed using a median value of each parameter. Parameters with low variance were ignored during the fine-tuning of each algorithm. Data of each parameter were scaled based on the interquartile range.

To evaluate the performance of each ML algorithm, accuracy, precision, recall, and F1–score were used. Accuracy, precision, recall, and F1–score are derived by equations shown below [47,59]:

(10) $$Accuracy=\left(TP+TN\right)/\left(TP+TN+FP+FN\right),$$

(11) $$Precision=TP/\left(TP+FP\right),$$

(12) $$Recall=TP/\left(TP+FN\right),$$

(13) $$F1-score=2\left\{\left(Recall\times Precision\right)/\left(Recall+Precision\right)\right\},$$

where TP denotes true positive (in case the ML model classifies an input as positive and the input is positive), TN denotes true negative (in case the ML model classifies an input as negative and the input is negative), FP denotes false positive (in case the ML model classifies an input as positive but the input is negative), and FN denotes false negative (in case the ML model classifies an input as negative but the input is positive).

Accuracy indicates how much an ML model correctly classifies the input. Precision and Recall trade off based on their equations. The numerator of Precision and Recall is TP while the denominators of Precision and Recall are TP+FP (all items classified as positive) and TP+FN (all positive items), respectively. F1−score, which considers both Precision and Recall simultaneously, is a harmonic mean of Precision and Recall. Each ML algorithm was run 600 times by randomly partitioning training and hold-out data (80% and 20%) to partially mitigate the issue of the limited datasets. Then, the average and 95% confidence intervals of accuracy, precision, recall, and F1–score were calculated.

## 3. Results

### 3.1. Behavioral Tests

**Figure 3:**
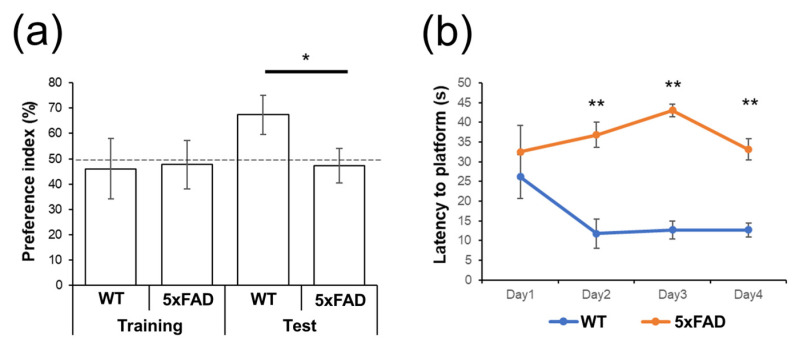
(**a**) Result of novel object recognition test in training and test phases; (**b**) result of Morris water maze as the day passes. All data show means ± standard error of the mean. The significance of differences between the groups was determined using the paired Student’s *t*-test. All values of *p* less than 0.05 and 0.005 were considered to be statistically significant and indicated using ‘*’ and ‘**’, respectively.

As shown in Figure 3a, the percentage of preference index for the novel object in the 5xFAD group was significantly lower than that of WT mice. The data show that object recognition memory was impaired in 5xFAD mice compared to WT mice. The results indicate that 5xFAD animals are impaired in their spatial learning. As shown in Figure 3b, 5xFAD mice had a significantly longer latency time to escape the platform from day two of the consecutive four days compared to WT mice. This observation indicates that 5xFAD animals are impaired in their memory. Collectively with the results of the novel object indication test, we confirm that the 5xFAD mice used in this work are impaired in terms of spatial learning and recognition memory, which are typical symptoms of AD.

### 3.2. Monte Carlo Simulation of Probing Depth

**Figure 4:**
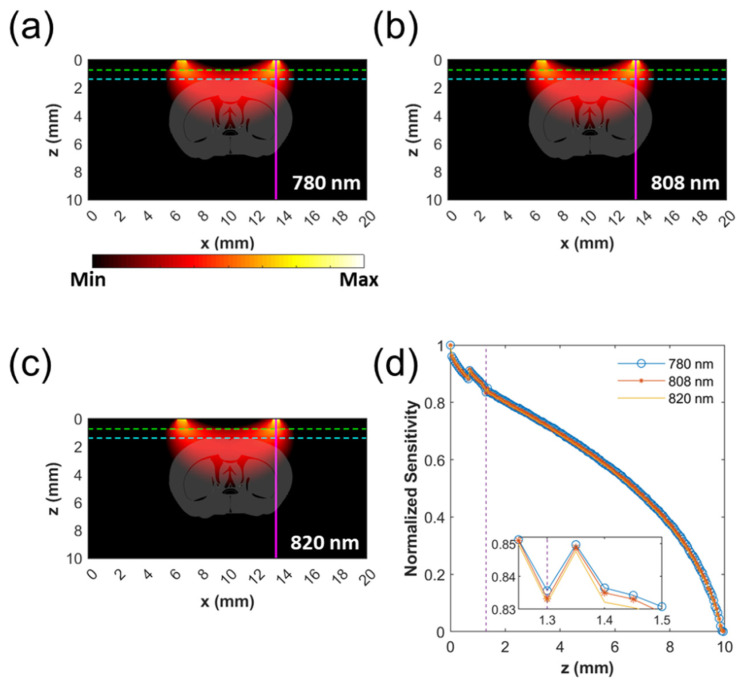
Depth sensitivity maps at wavelengths of 780 nm (**a**), 808 nm (**b**), and 820 nm (**c**) from the Monte Carlo simulation. In (**a**–**c**), green and cyan dotted lines represent boundaries between the scalp and the skull, and the skull and the brain, respectively. The probing depth is deep enough to reach parts of the cerebral cortex. (**d**) Normalized depth sensitivity through a magenta line of (**a**–**c**). A Purple dotted line represents a boundary (1.3 mm) between the skull and the brain. Inset shows a magnified image of normalized sensitivity at depths between 1.25 mm and 1.5 mm.

Figure 4 shows the results of Monte Carlo simulations. For better representation of the probing depth and region of the brain, depth sensitivity maps (Figure 4a–c) are superimposed on the mouse brain image that is based on a stereotaxic coordinate image of the mouse brain [60,61]. Due to minute changes in the optical properties of the tissues at different wavelengths, depth sensitivity maps at different wavelengths did not differ significantly. Similarly, normalized sensitivity along the depth did not change much as the wavelength varied (Figure 4d). At the interface between the skull and brain, depth sensitivity at 780 nm, 808 nm, and 820 nm remained to be 0.8357, 0.8332, and 0.8320, respectively. The maximum of depth sensitivity, which existed at the depth of 1.35 mm, of 780 nm, 808 nm, and 820 nm was 0.8497, 0.8448, and 0.8477, respectively. The results of the Monte Carlo simulations showed that more than 83% of photons could reach a depth deeper than 1.3 mm. In the meantime, more than 71% of photons could reach a 3.5 mm depth, which was the expected depth based on the diffusion theory. The sensitivity maps show that the light is mostly probing the cerebral cortex, thus the feasibility of non-invasive brain signal acquisition using the current setup is confirmed. Furthermore, due to the use of close wavelengths, the probing regions of different wavelengths are well overlapped.

### 3.3. Grand Average of Hemoglobin Concentration

**Figure 5:**
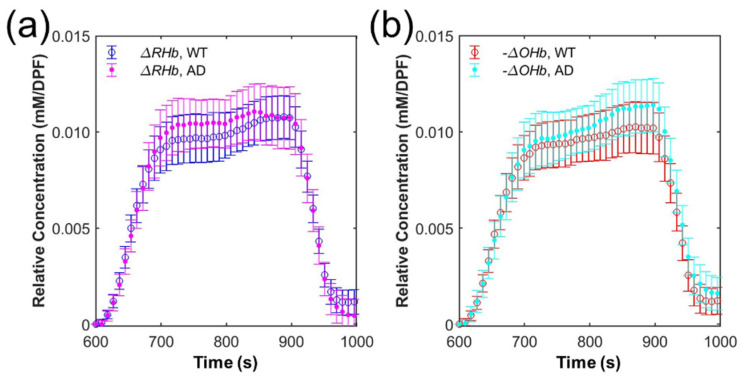
(**a**) Grand average and 95% confidence interval of relative deoxyhemoglobin concentration change (ΔRHb) of wild-type (WT) (blue circle) and Alzheimer’s disease (AD) (purple dot) mice. (**b**) Grand average and 95% confidence interval of the negative relative oxyhemoglobin concentration change (−ΔOHb) of WT (red circle) and AD (cyan dot).

Figure 5 shows the grand average of ΔRHb and −ΔOHb during hypoxic respiratory challenge. Hypoxic respiratory challenge was started at 600 s and ended at 900 s. In AD and WT, both ΔRHb and −ΔOHb showed a rapid increase until approximately 700 s (ΔRHb, AD: 0→0.00971 mM/DPF and WT: 0→0.00904 mM/DPF; −ΔOHb, AD: 0→0.00907 and WT: 0→0.00862) before showing a relatively slow increase or plateau until 900 s (ΔRHb, AD: 0.00971 mM/DPF →0.01072 mM/DPF and WT: 0.00904 mM/DPF→0.01065 mM/DPF; −ΔOHb, AD: 0.00907 mM/DPF→0.01138 mM/DPF and WT: 0.00862 mM/DPF→0.01012 mM/DPF). The increase of ΔRHb and −ΔOHb had a tendency of the double Exponential function (e.g., $A_{1}e^{r_{1}t}+A_{2}e^{r_{2}t}$). After the end of hypoxic respiratory challenge at 900 s, all of ΔRHb and −ΔOHb promptly decreased until approximately 960 s.

While ΔRHb and −ΔOHb showed similar trends in both WT and AD, they had some minute differences. ΔRHb of WT reached its maximum (0.01079 mM/DPF) at 887.1 s while the one of AD reached its maximum (0.01112 mM/DPF) at 845.3 s. −ΔOHb of WT reached its maximum (0.01030 mM/DPF, minimum of ΔOHb) at 866.3 s while the one of AD reached its maximum (0.01143 mM/DPF) at 891.2 s. Note that we analyzed −ΔOHb, not ΔOHb, in order to be consistent with the use of −ΔOHb in the analysis of the hemodynamic features.

### 3.4. Statistical Analysis

Table 2 and Table 3 show summaries of 13 hemodynamic features and their statistical analyses of ΔRHb and −ΔOHb, respectively. In ΔRHb, *A_rise,_*_2_ and *q_rise,_*_2_ showed *p*-values of 0.0086 and 0.0066, respectively. While *A_fall_* and *q_rise,1_* of ΔRHb showed quite a large difference in the mean between AD and WT, their *p*-values were greater than 0.05 (0.2798 and 0.8052). In −ΔOHb, *m_rise_* showed a *p*-value of 0.0499. Concurrently, *A_rise,_*_1_, *q_rise,_*_1_, and *S_max_* of −ΔOHb showed quite a large difference in the mean between AD and WT, even though their *p*-values were greater than 0.05 (0.2485, 0.1338, and 0.0566).

### 3.5. Machine Learning (ML)-Based Classification

The ML-based classification was performed on three different datasets: data with *p*-values lower than 0.05 (*A_rise,2_* and *q_rise,2_* of ΔRHb, and *m_rise_* of −ΔOHb), whole hemodynamic features of ΔRHb and −ΔOHb (23 hemodynamic features in total), and three principal components (PCs) from the whole hemodynamic features extracted via principal component analysis (PCA). PCA is a technique that is commonly used for the dimension reduction of multi-dimensional data to extract PCs with large variances [47,62]. The first PC is the extracted data with the largest variance, and the contribution of PCs to the variation of the data decreases as the order of PC increases (e.g., PC1 has a larger variance than PC2, PC2 has a larger variance than PC3, and so forth). Table 4 shows the classification results using data with *p*-values lower than 0.05. When data with *p*-values lower than 0.05 were used, logistic regression performed the best showing the best accuracy of 62.9%, followed by naive Bayes and quadratic discriminant analysis with an accuracy of 62.4%. Table 5 shows the classification results from the whole hemodynamic features. When the whole features were used, naive Bayes performed the best and showed the best accuracy of 84.3%. Table 6 shows the classification results using three PCs extracted from the whole hemodynamic features. When three PCs from the whole data were used, the extra trees classifier performed the best and showed the best accuracy of 76%. Among the best performing classifiers of different data sets (data with low *p*-values, whole hemodynamic features, and three PCs derived from the whole hemodynamic features), Naive Bayes using the whole hemodynamic feature performed the best in terms of accuracy. Logistic regression using data with low *p*-values performed the worst.

## 4. Discussion

In this work, the hypoxic respiratory challenge was used to induce hemodynamic reactivity in an animal model. Since animals cannot perform breath-holding spontaneously, the hypoxic respiratory challenge was used to mimic breath-holding even though hypoxic respiratory challenge may not be able to mimic breath-holding perfectly. The experimental protocol used in the work (50% O_2_ gas balanced by N_2_ gas for 10 min → 16% O_2_ gas balanced by N_2_ gas for 5 min as hypoxic gas challenge → 50% O_2_ gas balanced by N_2_ gas for 15 min) was based on a protocol used in a previous breast cancer study by S. Lee and J. G. Kim (100% O_2_ gas for 3 min → 21% O_2_ gas balanced by N_2_ gas for 10 min as the hypoxic gas challenge) with a minute modification [28]. In the previous study, the average pulmonary oxygen saturation (SpO_2_) of rats changed from 97% to 91% as the supplied gas was changed from 100% O_2_ gas to 21% O_2_ gas balanced by N_2_ gas. In this study, the average SpO_2_ changed from 98.6% to 94.4% as the supplied gas was changed from 50% O_2_ gas balanced by N_2_ gas to 16% O_2_ gas balanced by N_2_ gas. While the drop of SpO_2_ in this study was smaller (98.6% → 94.4%: 4.2% difference) than the one of the previous study (97% → 91%: 6% difference) [28], the drop of SpO_2_, which is observed in a human study during breath-holding [63], guarantees that the protocol used in this study can mimic breath-holding as well. At the same time, 94.4% SpO_2_ can assure the safety of the animal because this value is higher than the one (92% SpO_2_) of people living in Aspen, Colorado where people do not have any critical health issues [28]. The discrepancy between the study of S. Lee and J. G. Kim and this study may come from the use of different experimental protocols and the difference in animals used. While the protocol—either by letting a subject perform breath-holding spontaneously or supplying hypoxic gas—can be relatively easily carried out, the use of the protocol should be avoided for people with respiratory diseases.

As the ultimate goal of the suggested technique is to translate the technique to clinics in the future, the total amount of time for performing signal acquisition and processing—including the conversion of the raw data to hemoglobin concentration data and classification using an ML algorithm—should be short enough. In the animal experiments, assuming that all preparations—including shaving and depilating the fur on the leg and the head—are done, it takes 30 min for signal acquisition. The signal acquisition time can be shortened to several minutes maximum, which would include minutes of baseline and recovery measurements and breath holding or breathing of hypoxic gas if the signal acquisition is performed on human subjects. The conversion of optical intensity data to hemoglobin concentration and the extraction of hemodynamic features takes less than one minute, and may take a shorter length of time if the amount of the data becomes smaller (e.g., via the use of a shorter experimental protocol). For classification, it would take several seconds at maximum using a pre-trained ML algorithm. Overall, the total time may take less than 40 min for animals in this work and several minutes for humans. Thus, the signal acquisition and processing time of the technique will not be a hurdle when translating the technique to the clinics in the future.

Other than this work, there have been studies that attempted using breath-holding in mild cognitive impairment (MCI) and AD studies. Rather than attempting to extract various features from CVR signals, most of the previous MCI and AD studies that utilized transcranial Doppler for monitoring blood flow used the so-called breath-holding index, which is relatively simple [64,65]. Even though this work is not the first work that uses breath-holding for investigating AD or MCI, by using the simple DOS system for the acquisition of hemodynamic signals, extracting hemodynamic features from CVR caused by breath-holding, and adopting modern ML algorithms, this work attempted to examine an alternative approach for differentiating AD and WT. Thus, we believe that such a new approach will be able to encourage researchers to investigate the use of hemodynamic features from CVR signals caused by breath-holding or other types of breathing gas (e.g., carbogen) for other brain diseases that cause the impairment of the cerebrovasculature. In the meantime, changes in hemodynamic features have also been studied in animal studies to test cyanide toxicity [26,66], breast cancer studies [41], and other brain diseases such as Parkinson’s disease [67] and traumatic brain injury [68]. We believe that the suggested approach, with modifications, has the potential to be used in cases other than AD, such as the cases mentioned above.

In this work, the best ML result (84% accuracy) was achieved when the whole hemodynamic features were used as input features, and naive Bayes was employed for binary classification of AD and WT. As mentioned in the introduction, we note that some previous studies attempted to use AI, including ML and DL, for the classification of a sub-type of AD based on hemodynamic signals provoked by various cognitive tests. A study by D. Yang et al. adopted N-back, Stroop, and verbal fluency tasks as interventions to classify MCI patients and healthy controls [10]. A commercial, multi-channel functional near-infrared spectroscopy (fNIRS) was utilized to acquire hemoglobin concentration maps in the study. Using a convolutional neural network (CNN)-based AI algorithm, the researchers achieved 90% accuracy using the verbal fluency test, and achieved the best accuracy of 98.61% using the N-back test. Another study by D. Yang et al. attempted to use resting-state fNIRS signals acquired using the same commercial fNIRS system for the classification of MCI patients and healthy controls [69]. A connectivity map of ΔOHb and ΔRHb features extracted using various pre-trained CNN models (e.g., variations of VGG, Densenet, Alexnet, and Resnet networks) for transfer learning, time-series of ΔOHb, and ΔRHb were used as input features for ML classifiers, including linear discriminant analysis, support vector machine, and K-nearest neighbor. As the time-series signals were given as inputs, the best classification accuracy only reached 67% using linear discriminant analysis. Interestingly, because the features extracted using VGG19, a type of widely used CNN model, were used, the best accuracy of 95.81% could be achieved when the support vector machine was used as a classifier. In the meantime, one study by T. K. K. Ho et al. adopted oddball, 1-back, and verbal fluency tasks as interventions and attempted to perform classification of multi-stage AD with the inclusion of healthy controls, asymptomatic AD (a group of people with amyloid deposition but without cognitive impairment), prodromal AD (a group of people with MCI), and AD (a group of people with significant cognitive impairment due to AD) [12]. A lab-built, multi-channel fNIRS was utilized to acquire time-series hemoglobin concentration signals in the study. For classification, the researchers tested various ML (linear discriminant analysis, k-nearest neighbor, Gaussian naive Bayes, support vector machine, AdaBoost, random forest, and ensemble learning) and DL (simple neural network, 1D-CNN, long-short term memory (LSTM), gated recurrent units, and a combination of CNN and LSTM (CNN-LSTM)) algorithms. Among ML algorithms, using ΔOHb, ensemble learning achieved the best accuracy of 82.9%. Among DL algorithms, using relative total hemoglobin concentration (ΔTHb), which is calculated via summation of ΔOHb and ΔRHb (i.e., ΔTHb=ΔOHb+ΔRHb), CNN-LSTM achieved an accuracy of 87.7%. The studies used more advanced AI algorithms as either a classifier or a feature extractor and achieved a better accuracy than the current study. However, the best accuracy in this study outperformed the one in one study by D. Yang et al. [69] with ML classifiers as ΔOHb and ΔRHb were used as inputs (84% vs. 67%), even though the direct comparison between the study of D. Yang et al. and this work is not straightforward due to a few differences such as differences in subjects. Still, by comparing results from pure ML classifiers and a combination of AI algorithms, the study of D. Yang et al. [69] shows the potential of using advanced AI techniques to fully extract hidden information in hemodynamic signals. In this work, we could not test more advanced AI techniques due to the use of a single-channel system and limited datasets. A limited population is another factor that limited the use of more advanced AI techniques. In the future, a study using a multi-channel system and a larger population will need to be performed to more rigorously test the use of breath-holding or hypoxic respiratory challenge in AD.

**Figure 6:**
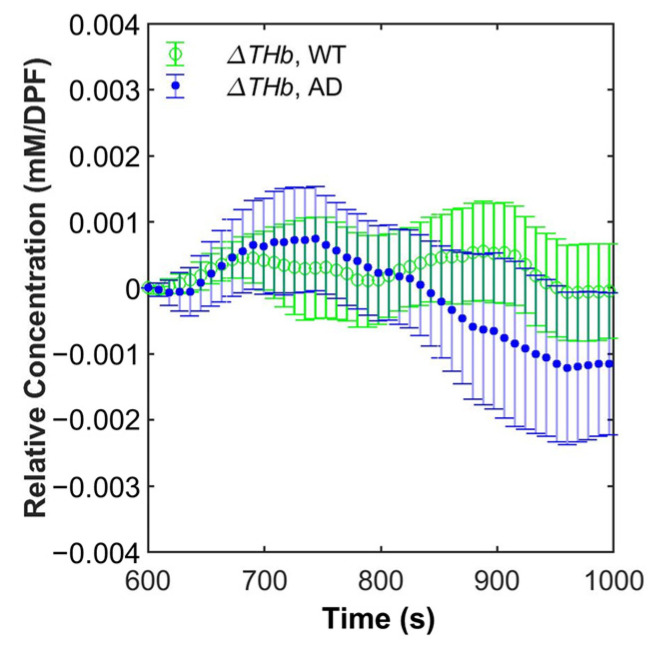
Grand average and 95% confidence interval of relative total hemoglobin concentration (ΔTHb) of WT (green circle) and AD (blue dot).

We postulated that CVR, which was measured using the DOS system, during breath-holding may be able to be used to differentiate AD and WT due to differences in the cerebrovasculature. While the time for onset and symptoms vary depending on AD animal models, many AD models show cerebrovascular abnormalities [21,22,70,71]. In the 5xFAD model, as β-amyloid accumulates, morphological abnormality of the cerebrovasculature, dysfunction of the blood–brain barrier, and variations in cerebrovascular density happen, which may be caused by or progressed due to inflammatory response and cerebral amyloid angiopathy [20,21,22,23]. In turn, neurovascular units (NVUs), which are responsible for maintaining homeostasis of the cerebrovasculature, are deteriorated and may fail their functions. As the deterioration of NVUs and the cerebrovasculature also happens in the 5xFAD model, we could observe CVR differences between AD and WT. ΔRHb in AD showed a larger maximum and took a shorter time to reach the maximum than ΔRHb in WT (AD: 0.01112 mM/DPF (845.3 s) and WT: 0.01079 mM/DPF (887.1 s)). Meanwhile, ΔOHb in AD showed a smaller minimum and took a longer time to reach the minimum than ΔOHb in WT (AD: −0.01143 mM/DPF (891.2 s) and WT: −0.01030 mM/DPF (866.3 s)). Additionally, as shown in Figure 6, starting from approximately 820 s, even after the end of hypoxic respiratory challenge at 900 s, ΔTHb in AD kept decreasing while ΔTHb in WT was maintained around the baseline level. The differences in ΔRHb, ΔOHb, and ΔTHb between AD and WT indicate that cerebrovascular impairment exists in the 5xFAD model. Such differences may originate from the impairments mentioned above, which can lead to the disruption of the regulation of cerebral blood flow and the exchange of O_2_ and CO_2_. We believe that these differences could be more quantitatively analyzed by utilizing 13 hemodynamic features. However, ML results using the whole hemodynamic features were better than the ones of other features used in this study. Since similar impairments happen in other AD models and even in clinical subjects [22,23,64,65,72], we expect that our technique can be translated to the clinics with some modifications.

As described above, this study has some limitations that should be addressed in the future. First, this study used a relatively small population. Even though each animal was measured multiple times to partially address the population issue, this approach may not fully resolve the issue of the limited population. In the meantime, the acquisition of signals from larger populations will be able to allow the use of more advanced AI techniques such as deep learning so that more advanced analyses, including multi-stage classification of AD, can be performed. Thus, a follow-up study with larger populations will need to be performed. Second, the DOS measurement was performed using a single source-detector separation. While the results of the Monte Carlo simulation guarantee the feasibility of the brain signal measurement using the DOS system, based on diffuse optics theories, deep tissue measurements of multi-layered tissues (e.g., the scalp, skull, and brain) are affected by the changes in optical properties of both extracerebral (e.g., the scalp and skull) and intracerebral tissues (e.g., the brain) [32]. To minimize the extracerebral signals, a multi-channel system with multiple source-detector pairs (e.g., with both short separation channels and long separation channels) can be used in the future [73]. Another advantage of using the multi-channel system includes the possibility of the acquisition of signals from multiple brain regions that can allow the use of more advanced analysis (e.g., functional connectivity) and more advanced AI techniques. Additionally, the use of a multi-modal system, including a combination of an optical system and an electroencephalogram [73], can be another interesting work that is worth investigating in the future. Furthermore, cytochrome c oxidase (CCO), which is closely related to the oxidative metabolism of mitochondria and the synthesis of adenosine triphosphate (ATP), can be another potential parameter that can be investigated due to the possibility of using DOS for CCO measurement [74] and the relationship between the decrease of CCO activity and the progress of AD [75,76]. Third, to perform a stable signal measurement, animals were anesthetized during the signal acquisition. While anesthesia is widely utilized in animal studies, the physiological response may be altered by anesthesia. In this study, hypoxic respiratory challenge, which was used to mimic breath-holding, was maintained for a relatively long time (5 min) to compensate the delayed response caused by anesthesia. However, this may not happen in human experiments. Rather, breath-holding is mostly performed for less than 1 min in human experiments. To mimic more realistic breath-holding in animal experiments, the animal can be placed in a container that is much larger than a usual induction chamber of anesthesia and different types of breathing gas can be supplied. In the meantime, a miniature, wireless system that can be fixed on the head can be used to monitor the brain response during breath-holding. Fourth, the best accuracy has not reached the level of similar studies. While the suggested method has the potential to be used in the early diagnosis of AD, there are many spaces where the method should be further improved to use the method on patients due to the simplicity of the method. Lastly, we only used a 5xFAD AD model and WT animals in this work. In the future, the same work may need to be performed with various types of AD models, including not only models of FAD but also models of sporadic AD, which is found to be about 95% of AD cases [22].

## 5. Conclusions

In conclusion, we performed a preclinical study to test the feasibility of combining hypoxic respiratory challenge, which was used to mimic breath-holding, and diffuse optical signal measurements to differentiate AD and WT. To quantify hemodynamic response during hypoxic respiratory challenge, we employed hemodynamic features that were originally used to study the hemodynamic response of breast cancer during breath-holding. To further confirm the usefulness of our approach, we used various ML techniques to classify AD and WT. Using whole hemodynamic features, the best accuracy of 84% was achieved. In the future, we will perform experiments with a larger population using multi-channel brain monitoring systems to investigate the applicability of the breath-holding protocol for classification of multi-stage AD.

## Figures and Tables

**Table 1:** Dimensional and optical properties used in Monte Carlo simulation. λ_1_: 780 nm; λ_2_: 808 nm; λ_3_: 820 nm.

	Scalp		Skull		Brain	
Thickness (mm) [36]	0.7		0.6		8.7	
Absorption coefficient (mm^−1^) [32,33,37]	λ_1_	0.0104	0.0250		λ_1_	0.0132
	λ_2_	0.0103			λ_2_	0.0131
	λ_3_	0.0107			λ_3_	0.0137
Scattering coefficient (mm^−1^) [18,32,36]	λ_1_	9.9982	λ_1_	25.2684	λ_1_	11.9932
	λ_2_	9.7269	λ_2_	24.6957	λ_2_	11.5491
	λ_3_	9.6257	λ_3_	24.4602	λ_3_	11.3683
Refractive index [32,38,39,40]	1.38		1.55		1.37	
Anisotropy [32,37,40]	0.80		0.92		0.90	

**Table 2:** Summary of 13 hemodynamic features of ΔRHb from WT and AD. std: standard deviation. *p*-value lower than 0.05 is highlighted using red color.

Features	WT (Mean ± std)	AD (Mean ± std)	*p*-Value	Both Passed Normality Test?
*IE*	1.03 × 10^0^ ± 1.68 × 10^−2^	1.03 × 10^0^ ± 3.53 × 10^−2^	0.8429	×
*PIE*	−4.00 × 10^−1^ ± 6.61 × 10^−2^	−3.94 × 10^−1^ ± 1.10 × 10^−1^	0.7950	○
*m_rise_*	3.63 × 10^−5^ ± 5.45 × 10^−6^	3.79 × 10^−5^ ± 1.46 × 10^−5^	0.6190	○
*m_fall_*	−1.43 × 10^−4^ ± 4.29 × 10^−5^	−1.61 × 10^−4^ ± 6.47 × 10^−5^	0.2389	○
*A_fall_*	6.31 × 10^4^ ± 8.89 × 10^4^	7.21 × 10^5^ ± 1.88 × 10^6^	0.2798	×
*A_rise,_*_1_	2.26 × 10^−2^ ± 3.70 × 10^−2^	3.81 × 10^−2^ ± 6.45 × 10^−2^	0.9298	×
*A_rise,_*_2_	−4.70 × 10^2^ ± 9.70 × 10^2^	−4.71 × 10^1^ ± 6.67 × 10^1^	0.0086	×
*q_fall_*	1.58 × 10^−2^ ± 4.09 × 10^−3^	1.79 × 10^−2^ ± 5.23 × 10^−3^	0.1143	○
*q_rise,_*_1_	−1.06 × 10^−3^ ± 2.84 × 10^−3^	−8.34 × 10^−4^ ± 3.77 × 10^−3^	0.8052	○
*q_rise,_*_2_	−1.62 × 10^−2^ ± 8.82 × 10^−3^	−9.68 × 10^−3^ ± 7.23 × 10^−3^	0.0066	○
*S_max_*	1.02 × 10^−2^ ± 1.87 × 10^−3^	−1.09 × 10^−2^ ± 4.51 × 10^−3^	0.4879	○
*S_min_*	−2.67 × 10^−4^ ± 1.81 × 10^−4^	−2.98 × 10^−4^ ± 2.60 × 10^−4^	0.6340	○
*S_PostBH30sec_*	6.00 × 10^−3^ ± 1.23 × 10^−3^	6.15 × 10^−3^ ± 2.56 × 10^−3^	0.8050	○

**Table 3:** Summary of 13 hemodynamic features of −ΔOHb from WT and AD. std: standard deviation. *p*-value lower than 0.05 is highlighted using red color.

Features	WT (Mean ± std)	AD (Mean ± std)	*p*-Value	Both Passed Normality Test?
*IE*	1.03 × 10^0^ ± 2.48 × 10^−2^	1.03 × 10^0^ ± 2.30 × 10^−2^	0.3207	×
*PIE*	−3.92 × 10^−1^ ± 1.10 × 10^−1^	−3.61 × 10^−1^ ± 1.34 × 10^−1^	0.3518	○
*m_rise_*	3.44 × 10^−5^ ± 8.60 × 10^−6^	4.04 × 10^−5^ ± 1.26 × 10^−5^	0.0499	○
*m_fall_*	−1.33 × 10^−4^ ± 4.14 × 10^−5^	−1.56 × 10^−4^ ± 6.14 × 10^−5^	0.1172	○
*A_fall_*	2.33 × 10^5^ ± 4.16 × 10^5^	3.35 × 10^5^ ± 1.02 × 10^6^	0.4202	×
*A_rise,_*_1_	2.09 × 10^0^ ± 4.61 × 10^0^	1.33 × 10^−2^ ± 1.28 × 10^−2^	0.2485	×
*A_rise,_*_2_	−3.56 × 10^2^ ± 5.98 × 10^2^	−4.47 × 10^2^ ± 8.82 × 10^2^	0.8004	×
*q_fall_*	1.65×10^−2^ ± 5.59×10^−3^	1.69 × 10^−2^ ± 6.79 × 10^−3^	0.8486	○
*q_rise,_*_1_	−1.88 × 10^−3^ ± 3.31 × 10^−3^	−6.45 × 10^−4^ ± 2.69 × 10^−3^	0.1338	○
*q_rise,_*_2_	−1.37 × 10^−2^ ± 6.79 × 10^−3^	−1.46 × 10^−2^ ± 7.35 × 10^−3^	0.6446	○
*S_max_*	9.81 × 10^−3^ ± 2.61×10^−3^	1.16 × 10^−2^ ± 3.83 × 10^−3^	0.0566	○
*S_min_*	−2.68 × 10^−4^ ± 2.45 × 10^−4^	−3.29 × 10^−4^ ± 2.38 × 10^−4^	0.3751	○
*S_PostBH30sec_*	6.07 × 10^−3^ ± 2.31 × 10^−3^	7.49 × 10^−3^ ± 3.04 × 10^−3^	0.0615	○

**Table 4:** Averaged accuracy, recall, precision, and F1−score and their 95% confidence interval (CI) of 15 machine learning (ML) methods using hemodynamic features with *p*-values lower than 0.05 (*A_rise,2_ and q_rise,2_ of* ΔRHb*, and m_rise_* of −ΔOHb) for classification of AD and WT. Each method was performed 600 times by shuffling the data due to limited data sets. The results and the name of the ML method with the best accuracy (logistic regression) are highlighted in red.

Method	$Accuracy\;\left(\%\right)$ [95% CI]	$Recall\;\left(\%\right)$ [95% CI]	$Precision\;\left(\%\right)$ [95% CI]	$F1–Score\;\left(\%\right)$ [95% CI]
Logistic regression	62.9 [62.0, 63.9]	35.8 [34.1, 37.4]	71.8 [69.1, 74.5]	45.1 [43.5, 46.8]
Ridge classifier	52.4 [51.2, 53.5]	30.0 [28.5, 31.5]	62.2 [59.8, 64.6]	35.9 [31.7, 37.1]
Linear discriminant analysis	53.8 [52.6, 55.1]	7.3 [6.4, 8.1]	37.6 [33.7, 41.5]	11.9 [10.6, 13.2]
K-nearest neighbor classifier	51.9 [51.1, 52.7]	18.3 [16.9, 19.8]	42.1 [38.8, 45.3]	22.3 [20.7, 23.9]
Support vector machine	57.2 [56.2, 58.2]	52.2 [50.0, 54.4]	55.1 [52.7, 57.4]	48.4 [46.4, 50.4]
Naive Bayes	62.4 [61.2, 63.7]	25.7 [24.5, 26.9]	84.2 [81.3, 87.1]	38.4 [36.8, 40.0]
Decision tree classifier	50.1 [48.9, 51.4]	0.6 [0.3, 1.0]	1.5 [0.6, 2.3]	0.8 [0.3, 1.2]
Gradient boosting classifier	46.0 [44.9, 47.2]	60.8 [58.4, 63.2]	51.8 [50.0, 53.6]	48.6 [47.4, 49.9]
Light gradient boosting machine	55.1 [54.2, 55.9]	12.7 [11.0, 14.5]	18.0 [15.5, 20.5]	14.7 [12.7, 16.7]
Random forest classifier	44.6 [43.7, 45.5]	41.1 [37.6, 44.6]	32.6 [29.9, 35.4]	28.1 [26.0, 30.3]
Quadratic discriminant analysis	62.4 [61.4, 63.4]	27.5 [26.3, 28.6]	79.0 [76.1, 81.9]	39.7 [38.2, 41.2]
Extreme gradient boosting	49.5 [48.4, 50.7]	63.0 [59.3, 66.8]	32.3 [30.1, 34.5]	41.3 [38.7, 43.9]
AdaBoost classifier	59.5 [58.2, 60.8]	24.7 [23.2, 26.2]	67.8 [64.3, 71.2]	34.4 [32.5, 36.4]
Extra trees classifier	38.6 [37.6, 39.6]	39.1 [35.5, 42.8]	16.2 [14.6, 17.7]	22.3 [20.2, 24.4]
CatBoost classifier	46.7 [45.2, 48.2]	26.7 [24.8, 28.6]	44.8 [41.8, 47.8]	28.5 [26.9, 30.1]

**Table 5:** Averaged accuracy, recall, precision, and F1−score and their 95% CI of 15 ML methods using hemodynamic features with whole hemodynamic features for classification of AD and WT. Each method was performed 600 times by shuffling the data due to limited data sets. The results and the name of the ML method with the best accuracy (naive Bayes) are highlighted in red.

Method	$Accuracy\;\left(\%\right)$ [95% CI]	$Recall\;\left(\%\right)$ [95% CI]	$Precision\;\left(\%\right)$ [95% CI]	$F1–Score\;\left(\%\right)$ [95% CI]
Logistic regression	71.8 [70.8, 72.8]	69.7 [68.3, 71.0]	65.3 [63.7, 66.9]	65.1 [64.0, 66.2]
Ridge classifier	49.7 [48.7, 50.6]	42.0 [40.3, 43.8]	42.9 [40.9, 44.9]	40.3 [38.7, 41.9]
Linear discriminant analysis	67.0 [65.8, 68.2]	28.0 [26.0, 30.0]	64.3 [60.6, 67.9]	34.0 [31.9, 36.1]
K-nearest neighbor classifier	75.1 [74.0, 76.1]	43.5 [41.3, 45.7]	72.4 [69.7, 75.1]	51.9 [49.6, 54.2]
Support vector machine	66.7 [65.5, 67.8]	69.3 [66.8, 71.7]	50.4 [48.3, 52.5]	56.9 [54.8, 59.0]
Naive Bayes	84.3 [83.8, 84.8]	9.7 [68.4, 71.1]	89.9 [88.4, 91.4]	75.4 [74.4, 76.3]
Decision tree classifier	52.7 [51.2, 54.2]	24.6 [22.7, 26.5]	25.6 [23.5, 27.7]	23.4 [21.6, 25.1]
Gradient boosting classifier	51.6 [50.1, 53.1]	52.7 [50.2, 55.1]	41.5 [39.4, 43.6]	42.8 [40.9, 44.7]
Light gradient boosting machine	54.2 [52.5, 56.0]	16.7 [14.1, 19.4]	13.9 [11.9, 15.9]	14.2 [12.1, 16.3]
Random forest classifier	54.0 [52.5, 55.5]	28.8 [26.2, 31.5]	27.3 [25.1, 29.5]	24.8 [22.8, 26.8]
Quadratic discriminant analysis	81.3 [80.6, 81.9]	75.4 [74.0, 76.8]	76.6 [75.3, 77.8]	74.0 [73.0, 75.0]
Extreme gradient boosting	39.5 [38.4, 40.5]	78.9 [76.1, 81.6]	34.6 [33.1, 36.1]	44.9 [43.1, 46.7]
AdaBoost classifier	59.1 [58.1, 60.2]	39.5 [37.1, 41.8]	43.2 [40.5, 45.9]	39.6 [37.3, 42.0]
Extra trees classifier	54.4 [53.0, 55.8]	19.7 [16.9, 22.5]	17.8 [15.3, 20.4]	13.2 [11.5, 14.9]
CatBoost classifier	49.1 [47.5, 50.7]	38.3 [36.0, 40.6]	39.2 [36.8, 41.6]	38.1 [35.8, 40.3]

**Table 6:** Averaged accuracy, recall, precision, and F1−score and their 95% CI of 15 ML methods using three principal components (PCs) of principal component analysis (PCA) extracted from whole hemodynamic features for classification of AD and WT. Each method was performed 600 times by shuffling the data due to limited data sets. The results and the name of the ML method with the best accuracy (extra trees classifier) are highlighted in red.

Method	$Accuracy\;\left(\%\right)$ [95% CI]	$Recall\;\left(\%\right)$ [95% CI]	$Precision\;\left(\%\right)$ [95% CI]	$F1–Score\;\left(\%\right)$ [95% CI]
Logistic regression	73.8 [72.9, 74.7]	83.2 [82.0, 84.4]	69.3 [68.0, 70.5]	74.1 [73.1, 75.0]
Ridge classifier	73.5 [72.6, 74.4]	83.2 [82.0, 84.4]	68.9 [67.6, 70.2]	73.9 [73.0, 74.8]
Linear discriminant analysis	73.7 [72.8, 74.6]	83.2 [82.0, 84.4]	69.1 [67.9, 70.4]	74.0 [73.1, 74.9]
K-nearest neighbor classifier	73.8 [72.8, 74.7]	80.9 [79.4, 82.5]	68.9 [67.5, 70.4]	72.9 [71.6, 74.1]
Support vector machine	71.1 [70.3, 71.9]	81.7 [80.2, 83.1]	65.5 [64.2, 66.9]	71.1 [69.9, 72.2]
Naive Bayes	73.3 [72.5, 74.1]	85.6 [84.4, 86.7]	67.8 [66.6, 69.0]	74.1 [73.3, 75.0]
Decision tree classifier	61.5 [60.2, 62.7]	37.3 [34.3, 40.3]	47.2 [44.1, 50.2]	37.8 [35.1, 40.5]
Gradient boosting classifier	72.4 [71.2, 73.5]	67.4 [64.8, 70.0]	69.1 [67.0, 71.2]	64.5 [62.4, 66.7]
Light gradient boosting machine	65.7 [64.3, 67.0]	41.2 [37.8, 44.5]	41.9 [38.7, 45.0]	39.2 [36.2, 42.3]
Random forest classifier	69.0 [67.7, 70.2]	58.3 [55.5, 61.2]	62.9 [60.5, 65.4]	56.3 [53.9, 58.7]
Quadratic discriminant analysis	72.7 [718, 73.6]	83.8 [82.6, 85.0]	67.6 [66.4, 68.9]	73.4 [72.5, 74.3]
Extreme gradient boosting	63.1 [62.0, 64.1]	73.6 [71.4, 75.8]	60.1 [58.4, 61.7]	62.0 [60.5, 63.6]
AdaBoost classifier	65.2 [63.9, 66.5]	49.1 [46.1, 52.1]	56.2 [53.3, 59.1]	47.9 [45.3, 50.5]
Extra trees classifier	76.0 [75.1, 76.9]	80.1 [78.5, 81.7]	72.3 [70.9, 73.7]	74.2 [73.0, 75.4]
CatBoost classifier	65.8 [64.6, 67.0]	53.4 [50.5, 56.3]	60.5 [57.9, 63.1]	57.1 [49.3, 54.1]

## Data Availability

The data that support the findings of this study are available from the corresponding author upon reasonable request.
